# Prognostic value of monocyte-to-lymphocyte ratio for 90-day all-cause mortality in type 2 diabetes mellitus patients with chronic kidney disease

**DOI:** 10.1038/s41598-023-40429-6

**Published:** 2023-08-12

**Authors:** Chuangye Qiu, Shizhen Liu, Xingai Li, Wenxia Li, Guoqiang Hu, Fanna Liu

**Affiliations:** 1 Department of Nephrology, Jiangmen Central Hospital, Affiliated Jiangmen Hospital of Sun Yat-Sen University, Jiangmen, Guangdong China; 2 Department of Endocrinology, Jiangmen Central Hospital, Affiliated Jiangmen Hospital of Sun Yat-Sen University, Jiangmen, Guangdong China; 3 Department of Nephrology, The First Affiliated Hospital of Jinan University, Guangzhou, 510630 Guangdong China

**Keywords:** Biomarkers, Diseases, Endocrinology, Nephrology, Risk factors

## Abstract

The role of inflammation and the correlation between inflammatory markers and type 2 diabetes mellitus (T2DM) and chronic kidney disease (CKD) have been studied. In clinical work, a large number of T2DM patients complicated with CKD, but the cause of CKD was not clear. Our study aimed to evaluate the relationship between monocyte-to-lymphocyte ratio (MLR) and mortality in T2DM patients with CKD. The data from Medical Information Mart for Intensive Care III was analyzed. The primary outcome was 90-day all-cause mortality; the secondary outcomes were the length of ICU stay, hospital mortality and 30-day all-cause mortality. Cox regression was used to evaluate the association between MLR and 90-day mortality. We performed subgroup analyses to determine the consistency of this association, and used Kaplan–Meier survival curve to analysis the survival of different levels of MLR. A total of 1830 patients were included in study retrospectively. The length of ICU stay, 30-day all-cause mortality, and 90-day all-cause mortality in the MLR > 0.71 group were significantly higher than those in the MLR < 0.28 and 0.28 ≤ MLR ≤ 0.71 group. In Cox regression analysis, high MLR level was significantly associated with increased greater risk of 90-day all-cause mortality. The adjusted HR (95%CIs) for the model 1, model 2, and model 3 were 2.429 (1.905–3.098), 2.070 (1.619–2.647), and 1.898 (1.478–2.437), respectively. Subgroup analyses also showed the consistency of association between MLR and 90-day all-cause mortality. The Kaplan–Meier survival curve analysis revealed that MLR > 0.71 had worst prognosis. In T2DM patients with CKD in the intensive care unit, high MLR was significantly associated with increased risk 90-day all-cause mortality.

## Introduction

A study predicts that the global population of diabetes will increase to 783 million by 2045. The study found that the prevalence of chronic kidney disease (CKD) among diabetic patients in the United States during 2015–2020 was still high (64–81.6/1000), and with the significant increase of the prevalence of diabetes, the prevalence of CKD will be further increased^1^. Diabetes mellitus is the main cause of CKD and the main risk factor for progression from CKD to end stage renal disease^2^. A study showed that type 2 diabetes patients with CKD reduced life expectancy by 16 years than that of patients without CKD, and resulted in higher medical cost burden and mortality^3,4^. In patients with type 2 diabetes mellitus (T2DM) and CKD, the body is in an inflammatory state, and monocyte infiltration is stimulated by intermediate products such as advanced glycation end-products and immune complexes to aggravate cell damage, thus accelerating disease deterioration^5,6^.

Inflammation usually causes monocytes increased and lymphocytes decreased. Studies found that high monocyte and low lymphocyte values were positively associated with increased cardiovascular disease and mortality^7,8^. Monocyte to lymphocyte ratio (MLR) can reflect the degree of inflammation better than only high monocytes and low lymphocytes. High MLR was found to be significantly associated with increased risk of adverse cardiovascular outcomes in patients with atherosclerosis and could be used as a prognostic indicator of the degree of coronary artery stenosis^9^. Further, another study demonstrated that MLR was significantly associated with increased risk of the incidence of acute kidney injury in intensive care unit (ICU) patients and may become an effective biomarker^10^. Besides, a study showed that high MLR was an independent risk factor for stroke associated pneumonia and had predictive value for the severity of pneumonia in stroke associated pneumonia patients^11^.

In clinical work, a large number of T2DM patients complicated with CKD, but the cause of CKD was not clear. Patients were complicated with more underlying diseases in ICU, such as T2DM, CKD, hypertension and others, but we often ignored these problems in treatment, because there was lack of clinical prognostic research on T2DM patients complicated with CKD. The body of patients with T2DM and CKD was in a state of micro inflammation. As a new inflammatory indicator, MLR had also been confirmed to be related to the prognosis of ICU patients. Therefore, in our study, we explored the relationship between MLR and 90-day all-cause mortality of T2DM patients with CKD in ICU. We hope to provide doctors with reliable data related to the prognosis of T2DM patients with CKD.

## Materials and methods

### Study design

We obtained data from the Medical Information Mart for Intensive Care III (MIMIC-III) that contained information about 50,000 patients, respectively^12^. First, we selected patients according to the following inclusion and exclusion criteria. Second, we extracted demographic information, clinical laboratory data and related scoring information from the database. According to the interquartile ranges (IQRs) of MLR value, we divided into three groups: MLR < 0.28, 0.28 ≤ MLR ≤ 0.71 and MLR > 0.71. Then, we used Cox regression to develop a prediction model for the 90-day mortality. And subgroup and Kaplan–Meier analysis were used to evaluate the differences in different MLR levels in 90 day mortality of patients.

### Inclusion and exclusion criteria

**Figure 1:**
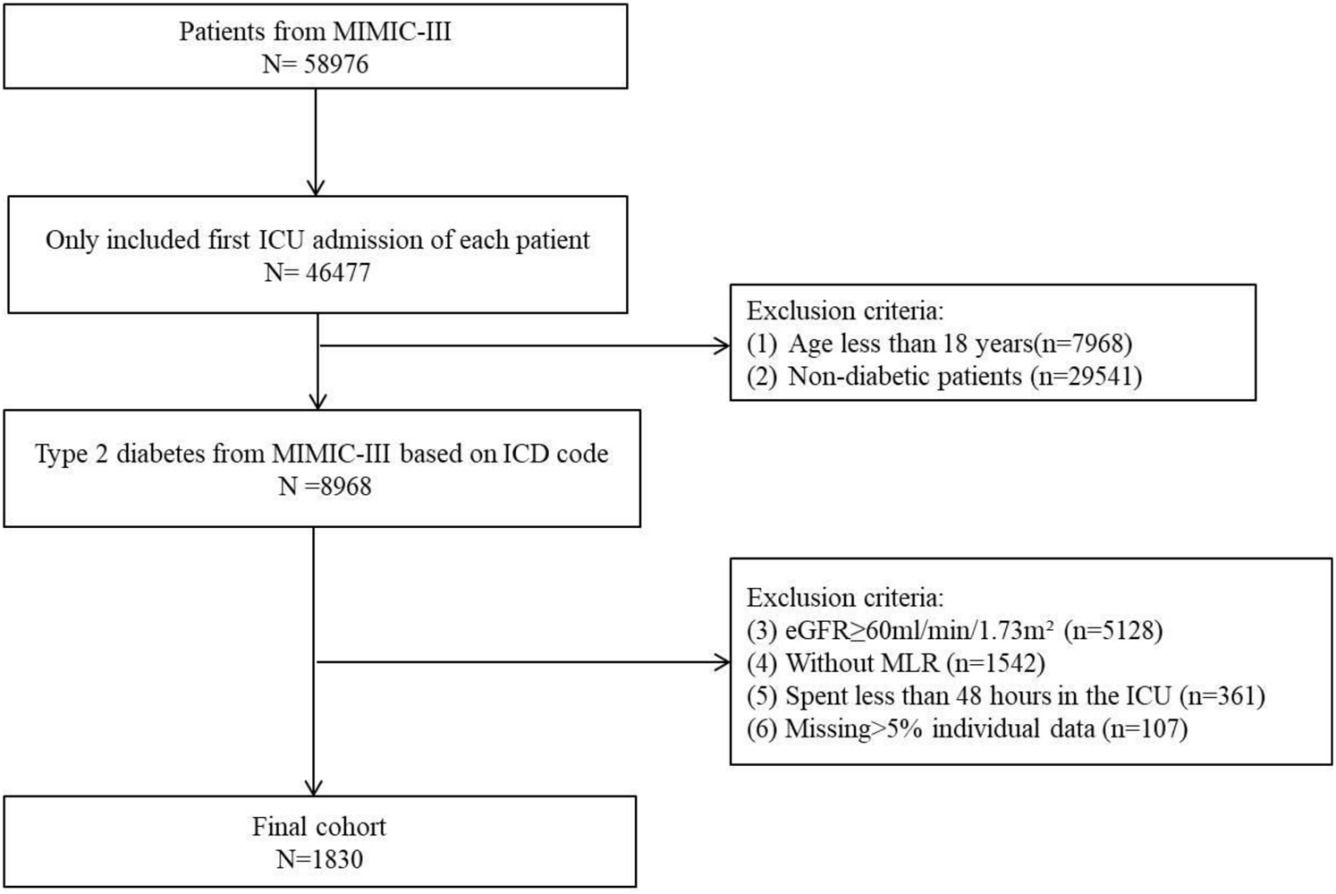
Flow diagram of the study.

Patients were selected according to the following inclusion and exclusion criteria. Inclusion criteria in our study: (1) patients admitted to the ICU for the first time; (2) ICD code is T2DM. Exclusion criteria: (1) less than 18 years of age; (2) non-type 2 diabetes mellitus; (3) without CKD, CKD was diagnosed that glomerular filtration rate (GFR) below 60 mL/min/1.73 m^2^ for 3 months or more^13^. (4) Missing value of lymphocyte and monocyte; (5) less than 48 h in ICU; (6) missing data for more than 5% of patients. Finally, 1830 patients were included in this study (Fig. 1).

### Data extraction

We extracted the variables from the database with structured query language in PostgreSQL, including age, gender, weight, coronary artery disease (CAD), chronic heart failure (CHF), hypertension, CKD stage, sequential organ failure assessment (SOFA), hemoglobin (HGB), platelet, monocytes, lymphocytes, potassium, sodium, phosphate, creatinine, blood urea nitrogen (Bun), albumin (ALB), alkaline phosphatase (ALP), alanine transaminase (ALT), aspartate transaminase (AST), glucose, Prothrombin time (PT), partial thromboplastin time (PTT) and estimate-GFR (eGFR). The formula of GFR estimation was

$$ {186}\, \times \,{\text{serum }}\;{\text{creatinine}}\, - \,{1}.{154}\, \times \,{\text{age}}\, - \,0.{2}0{3}\, \times \,0.{742 }\left( {{\text{females}}} \right)\, \times \,{1}.{233}. $$

### Ethics approval and consent to participate

The informed consent was waived by the Institutional Review Boards of Beth Israel Deaconess Medical Center (Boston, MA) and the Massachusetts Institute of Technology (Cambridge, MA). The patient’s information has been standardized and the project did not affect clinical care, so requirement for individual patient consent was waived.

## Statistical analysis

Continuous variables were expressed as mean ± standard deviations or IQRs, and classification variables were expressed as totals and percentage (%). The chi-square test was utilized to compare classified variables between groups. The Wilcoxon rank-sum test was used for non-normally distributed continuous variables. The student t-test and analysis of variance were used for normally distributed continuous variables. Simple and multivariable Cox regressions were used to identify predictors of 90-day all-cause mortality, which expressed as the hazard ratio (HR) and 95% confidence interval (CI). In order to reduce the impact of confounding factors, we constructed three Cox regression models by including covariates with p values < 0.05 in the simple Cox analyses or for importance of clinical concern. We brought the MLR into the cox model in the form of every 0.1 increase in MLR. Kaplan–Meier survival analysis was used to determine the difference MLR level in 90-day all-cause mortality between the three groups. We performed stratification analyses to assess the association of MLR with 90-day all-cause mortality across various subgroups classified by age, gender, hypertension, CHF, CAD, SOFA score, weight, CKD stage, phosphate, Bun, ALB, ALT, ALP and PTT. p < 0.05 was considered statistically significant. The statistical analyses were conducted by using the Stata software version 16.0 (Stata Corp. LLC, TX, US).

## Results

### Baseline characteristics

According to the MLR, 1830 patients were enrolled and were divided into the three groups: 454 patients were in the low-MLR group (MLR < 0.28), 919 patients were in the mid-MLR group (0.28 ≤ MLR ≤ 0.71), and 457 patients were in the high-MLR group (MLR > 0.71). Patients with higher MLR were trend to be male, higher weight and CKD 4 stage; Moreover, these patients had higher SOFA score, potassium, phosphate, creatinine, Bun, ALP and PT; They also had lower sodium and eGFR (all p < 0.05) (Table 1).

**Table 1:** Comparisons of demographics within three MLR levels.

Variable	MLR < 0.28 (n = 454)	MLR 0.28–0.71 (n = 919)	MLR > 0.71 (n = 457)	p value
Age (years)	75.4 (66.4, 82.1)	76.9 (68.0, 83.7)	75.7 (67.4, 83.4)	0.175
Male, n (%)	207 (45.6)	476 (51.8)	261 (57.1)	0.002
Weight (kg)	76.2 (66.0, 88.1)	78.3 (65.4, 90.9)	79.7 (66.4, 92.5)	0.088
Comorbidities, n (%)				
CAD	131 (28.9)	346 (37.6)	146 (31.9)	0.003
CHF	195 (43.0)	463 (50.4)	228 (49.9)	0.027
Hypertension	171 (37.7)	331 (36.0)	147 (32.2)	0.073
CKD stage, n (%)				0.008
3	295 (65.0)	569 (61.9)	252 (55.1)	
4	103 (22.7)	242 (26.3)	152 (33.3)	
5	56 (12.3)	108 (11.7)	53 (11.6)	
SOFA score	5.0 (3.0, 7.0)	5.0 (3.0, 7.0)	6.0 (4.0, 9.0)	< 0.001
Laboratory tests				
HGB (× g/L)	10.3 (8.9, 11.4)	10.3 (9.2, 11.5)	10.0 (9.0, 11.5)	0.555
Platelet (× 10^9^/L)	202.0 (151.0, 280.0)	210.0 (154.0, 279.0)	207.0 (142.0, 277.0)	0.702
Monocytes (× 10^9^/L)	0.31 (0.18, 0.47)	0.48 (0.35, 0.69)	0.72 (0.48, 1.10)	< 0.001
Lymphocytes (× 10^9^/L)	1.60 (1.01, 2.36)	1.12 (0.78, 1.57)	0.60 (0.37, 0.95)	< 0.001
Potassium (mmol/L)	4.3 (3.8, 4.8)	4.3 (3.8, 4.9)	4.4 (3.9, 4.9)	0.034
Sodium (mmol/L)	138.7 ± 6.7	138.6 ± 5.1	137.7 ± 5.2	0.003
Phosphate (mg/dL)	4.0 ± 1.4	4.2 ± 1.6	4.5 ± 1.8	< 0.001
Creatinine (mg/dL)	1.8 (1.3, 2.9)	1.9 (1.3, 3.1)	2.1 (1.4, 3.4)	0.005
Bun (mg/dL)	42.0 ± 24.7	45.9 ± 24.6	50.9 ± 27.9	< 0.001
ALB (g/dL)	2.9 (2.8, 3.2)	2.9 (2.8, 3.2)	2.9 (2.6, 3.1)	0.166
ALP (U/L)	121.5 (74.0, 125.4)	120.0 (72.0, 125.4)	125.0 (80.0, 136.0)	0.024
ALT (IU/L)	43.0 (19.0, 105.7)	45.0 (18.0, 105.7)	47.0 (20.0, 105.7)	0.792
AST (IU/L)	59.5 (27.0, 166.4)	62.0 (27.0, 166.4)	64.0 (29.0, 166.4)	0.603
Glucose(mg/dL)	184.7 ± 105.4	180.4 ± 95.3	174.6 ± 87.2	0.285
PT (s)	14.2 (13.0, 16.0)	14.6 (13.4, 17.0)	15.1 (13.5, 17.9)	< 0.001
PTT (s)	39.5 ± 25.6	41.5 ± 25.5	42.8 ± 28.4	0.015
eGFR (ml/min/1.73 m^2^)	36.9 (23.3, 49.3)	35.3 (23.3, 46.9)	33.0 (21.5, 44.1)	0.010

MLR, monocyte-to-lymphocyte ratio; CAD, coronary artery disease; CHF, chronic heart failure; CKD, chronic kidney disease; SOFA, sequential organ failure assessment; HGB, hemoglobin; Bun, blood urea nitrogen; ALB, albumin; ALP, alkaline phosphatase; ALT, alanine transaminase; AST, aspartate transaminase; PT, prothrombin time, PTT partial thromboplastin time, eGFR estimated GFR.

### MLR levels and outcome

Among the three levels of MLR, the length of stay, in-hospital mortality, 30-day all-cause mortality and 90-day all-cause mortality in the MLR > 0.71 group were significantly higher than those in the MLR < 0.28 and 0.28 ≤ MLR ≤ 0.71 group (p < 0.05) (Table 2).

**Table 2:** MLR level and clinical outcome.

Clinical outcomes	MLR			
	< 0.28 (n = 454)	0.28–0.71 (n = 919)	> 0.71 (n = 457)	p value
Length of stay (days)	2.9 (1.7, 5.3)	3.0 (1.7, 5.9)	5.0 (3.8, 8.1)	< 0.001
Hospital mortality (n, %)	54 (11.9)	134 (14.6)	113 (24.7)	< 0.001
30-day mortality (n, %)	79 (17.4)	204 (22.2)	156 (34.1)	< 0.001
90-day mortality (n, %)	96 (21.1)	275 (29.9)	201 (44.0)	< 0.001

MLR, monocyte-to-lymphocyte ratio.

### Association between the MLR and 90-day all-cause mortality

Simple analysis showed that age, male, CAD, CKD stage, SOFA score, phosphate, Bun, ALB, ALP, PTT, eGFR, MLR, mid-MLR and high-MLR were significantly associated with 90-day all-cause mortality (p < 0.05) (Table 3). Adjust for age, gender, weight, CAD, CHF, hypertension and SOFA score in model 2, high MLR level was significantly associated with increased greater risk of 90-day all-cause mortality (HR 2.070, 95% CI 1.619–2.647, p < 0.05). Furthermore, Adjust for model 2 plus CKD stage, HGB, platelet, potassium, sodium, phosphate, Bun, ALB, ALP, ALT, AST, glucose, PT, PTT and eGFR in model 3, high MLR level remained a greater risk of 90-day all-cause mortality (HR 1.898, 95% CI 1.478–2.437, p < 0.05) (Table 4).

**Table 3:** Simple Cox regression analyses to assess risk factors associated with 90-day mortality in T2DM patients with CKD.

	HR (95%CI)	p value
Age	1.026 (1.018–1.034)	< 0.001
Male	0.780 (0.661–0.921)	0.003
Weight	0.996 (0.992–0.999)	0.029
CAD	0.794 (0.664–0.949)	0.011
CHF	1.121 (0.951–1.320)	0.173
Hypertension	0.977 (0.823–1.160)	0.792
CKD stage	1.175 (1.053–1.312)	0.004
SOFA score	1.116 (1.090–1.141)	< 0.001
HGB	1.008 (0.963–1.055)	0.746
Platelet	1.000 (0.999–1.001)	0.236
Potassium	1.005 (0.914–1.106)	0.911
Sodium	1.006 (0.991–1.022)	0.422
Phosphate	1.077 (1.027–1.130)	0.002
Creatinine	0.985 (0.994–1.028)	0.493
Bun	1.007 (1.004–1.010)	< 0.001
ALB	0.601 (0.506–0.713)	< 0.001
ALP	1.001 (1.000–1.002)	< 0.001
ALT	1.000 (0.999–1.001)	0.169
AST	1.000 (0.999–1.001)	0.063
Glucose	0.999 (0.998–1.000)	0.232
PT	1.008 (0.998–1.018)	0.106
PTT	1.005 (1.002–1.007)	0.001
eGFR	0.988 (0.983–0.994)	< 0.001
MLR, per 0.1	1.016 (1.011–1.022)	< 0.001
MLR < 0.28	Ref.	–
0.28 ≤ MLR ≤ 0.71	1.472 (1.167–1.857)	0.001
MLR > 0.71	2.429 (1.905–3.098)	< 0.001

MLR, monocyte-to-lymphocyte ratio; CAD, coronary artery disease; CHF, chronic heart failure; CKD, chronic kidney disease; SOFA, sequential organ failure assessment; HGB, hemoglobin; Bun, blood urea nitrogen; ALB, albumin; ALP, alkaline phosphatase; ALT, alanine transaminase; AST, aspartate transaminase; PT, prothrombin time; PTT, partial thromboplastin time; eGFR, estimated GFR, Ref reference.

**Table 4:** Association between MLR and 90-day mortality.

Variables	Model 1		Model 2		Model 3	
	HR (95% CI)	p value	HR (95% CI)	p value	HR (95% CI)	p value
MLR, per 0.1	1.016 (1.011–1.022)	< 0.001	1.011 (1.004–1.018)	0.001	1.009 (1.002–1.016)	0.010
MLR < 0.28	Ref.	–	Ref.	–	Ref.	–
0.28 ≤ MLR ≤ 0.71	1.472 (1.167–1.857)	0.001	1.449 (1.147–1.830)	0.002	1.397 (1.105–1.765)	0.005
MLR > 0.71	2.429 (1.905–3.098)	< 0.001	2.070 (1.619–2.647)	< 0.001	1.898 (1.478–2.437)	< 0.001

MLR, monocyte-to-lymphocyte ratio; Ref, reference; HR, hazard ratio; CI, confidence interval. Model 1: unadjusted. Model 2: adjust for: age; gender; weight; CAD; CHF; hypertension; and SOFA score. Model 3; adjust for: Model 2 plus CKD stage; HGB; platelet; potassium; sodium; phosphate; Bun; ALB; ALP; ALT; AST; glucose; PT; PTT and eGFR.

### Subgroup analyses

We performed subgroup analyses to determine the consistency of association between MLR and risk of 90-day all-cause mortality (Table 5). We found that patients with age < 65(HR 2.090, 95% CI 1.525–2.864), man (HR 1.573, 95% CI 1.340–1.848), without hypertension (HR 1.635, 95% CI 1.407–1.899), CHF (HR 1.618, 95% CI 1.364–1.920), CAD (HR 1.599, 95% CI 1.271–2.011), SOFA score (HR 1.909, 95% CI 1.518–2.400), weight ≥ 78 (HR 1.612, 95% CI 1.353–1.919), CKD 4 stage (HR 1.565, 95% CI 1.274–1.922), phosphate ≥ 4 (HR 1.593, 95% CI 1.361–1.834), Bun ≥ 4 (HR 1.671, 95% CI 1.419–1.969), ALB ≥ 2.9 (HR 1.695, 95% CI 1.451–1.979) and ALP ≥ 122 (HR 1.733, 95% CI 1.460–2.058) had a significantly higher risk of 90-day all-cause mortality with high MLR level.

**Table 5:** Subgroup analysis of the associations between MLR and 90-day mortality.

Subgroup	HR (95%CI)	p value
Age, years		
< 65	2.090 (1.525–2.864)	< 0.001
≥ 65	1.492 (1.310–1.699)	< 0.001
Gender		
F	1.541 (1.288–1.843)	< 0.001
M	1.573 (1.340–1.848)	< 0.001
Hypertension		
No	1.635 (1.407–1.899)	< 0.001
Yes	1.479 (1.212–1.803)	< 0.001
CHF		
No	1.529 (1.293–1.807)	< 0.001
Yes	1.618 (1.364–1.920)	< 0.001
CAD		
No	1.569 (1.366–1.803)	< 0.001
Yes	1.599 (1.271–2.011)	< 0.001
SOFA score		
< 5	1.909 (1.518–2.400)	< 0.001
≥ 5	1.392 (1.211–1.600)	< 0.001
Weight		
< 78	1.563 (1.327–1.842)	< 0.001
≥ 78	1.612 (1.353–1.919)	< 0.001
CKD stage		
3	1.557 (1.323–1.832)	< 0.001
4	1.565 (1.274–1.922)	< 0.001
5	1.406 (0.993–1.991)	0.055
Phosphate		
< 4	1.484 (1.233–1.786)	< 0.001
≥ 4	1.593 (1.361–1.834)	< 0.001
Bun		
< 40	1.428 (1.197–1.705)	< 0.001
≥ 40	1.671 (1.419–1.969)	< 0.001
ALB		
< 2.9	1.339 (1.112–1.612)	< 0.001
≥ 2.9	1.695 (1.451–1.979)	< 0.001
ALP		
< 122	1.439 (1.218–1.701)	< 0.001
≥ 122	1.733 (1.460–2.058)	< 0.001

HR, hazard ratio; CI, confidence interval; CAD, coronary artery disease; CHF, chronic heart failure; CKD, chronic kidney disease; SOFA, sequential organ failure assessment; Bun, blood urea nitrogen; ALB, albumin; ALP, alkaline phosphatase.

### Kaplan–Meier analysis

**Figure 2:**
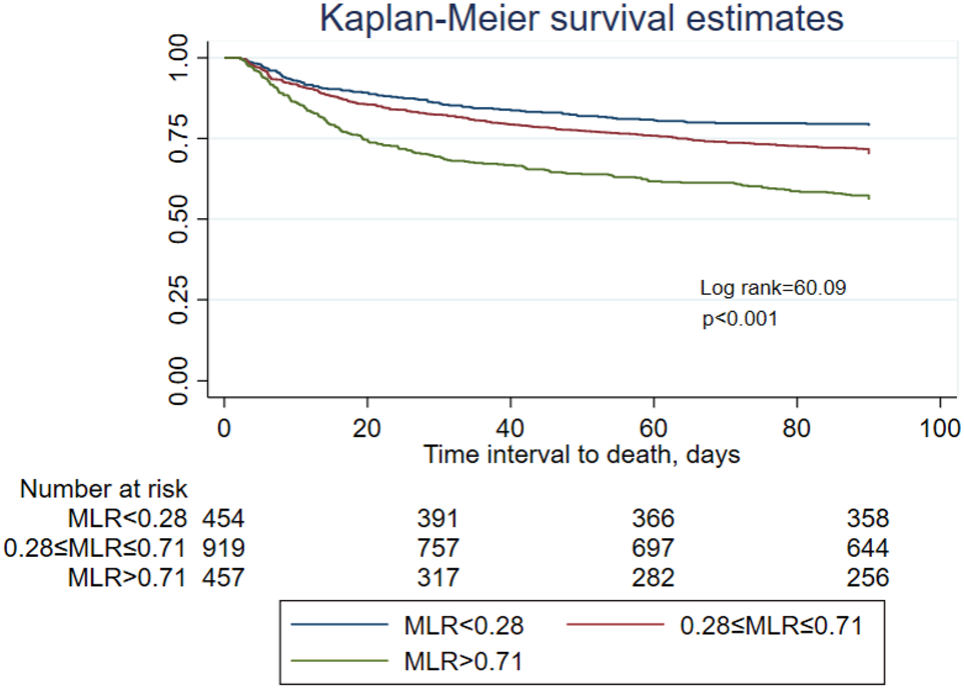
Kaplan–Meier curve was used to evaluate the difference between MLR levels and 90-day all-cause mortality in T2DM patients with CKD in the ICU. In the Kaplan–Meier analysis, the log-rank test p value < 0.001.

The patients were divided into three groups based on MLR level. The Kaplan–Meier survival curve analysis revealed that MLR > 0.71 had worst prognosis. Patients in higher MLR group had significantly higher 90-day all-cause mortality than low and mid-MLR groups (MLR > 0.71 vs 0.28 ≤ MLR ≤ 0.71 vs MLR < 0.28; 44% vs 29.9% vs 21.1%, respectively; log-rank test p value < 0.001) (Fig. 2).

## Discussion

Inflammation could be divided into infectious and non-infectious inflammation. Traditional inflammatory markers such as C-reactive protein and procalcitonin indicate the degree of infectious inflammation^14,15^. However, non-infectious inflammation leads to traditional inflammatory markers significantly individual differences, thus affecting its efficacy. Both infectious and non-infectious inflammation, the body’s immune regulation causes lymphocyte apoptosis and monocyte proliferation to increase, thereby exerting immune effects^16,17^. When T2DM and CKD are in a state of chronic inflammation, the lymphocytes and monocytes in the blood system will undergo subtle changes, which may be closely related to the prognosis of patients. Our study was the first study to demonstrate a new inflammatory marker that MLR was association with 90-day all-cause mortality in T2DM patients with CKD in the ICU.

MLR had been used as a biomarker of inflammation in various studies due to its ease of obtaining from blood parameters^9,18,19^. It has been reported that elevated MLR could serve as independent risk factors for multiple infections, autoimmune diseases, acute and chronic cardiovascular events, and cancer progression^20–23^. Studies showed that MLR was significantly higher than in patients with diabetic retinopathy and could be an independent risk factor for the progression of diabetic retinopathy^24,25^. Besides, the value of MLR in microalbuminuria group was higher than normoalbuminuria group in T2DM, it showed that MLR could be used as a predictor of kidney injury in T2DM^6^. Furthermore, an increased MLR was associated with higher mortality in patients with CKD^26^. This is consistent with our finding. Our study also showed that high MLR level increased the risk of 90-day mortality in T2DM patients with CKD in the ICU. A study included 355 maintenance hemodialysis patients found that MLR was an independent risk factor for poor prognosis^27^. Another study also found that MLR was a strong predictor of all-cause and cardiovascular death in hemodialysis patients^28^. Similarly, our study demonstrated that MLR was an independent risk factor for 90-day all-cause mortality in T2DM patients with CKD. (HR 1.178, 95% CI 1.110–1.249).

A multicenter retrospective cohort study showed that MLR > 0.45 was significantly associated with HR for CVD mortality of 1.45 at the commencement of peritoneal dialysis^29^. And a study also showed that higher MLR is associated with increased risks of both CVD events and infectious disease hospitalization in dialysis patients^30^. Similarly, in our study, we also found that the higher MLR group had higher length of stay in the ICU, hospital mortality, 30-day and 90-day all-cause mortality. Furthermore, the Kaplan–Meier survival curve analysis revealed that MLR > 0.71 had worst prognosis. After adjusting for confounding variables, higher MLR was also found to be a predictor of increased atherosclerosis in patients with diabetes^31^. Similarly, we controlled for confounding variables by multivariable Cox regression analysis and also found that high MLR was an independent risk factor for 90-day mortality. In addition, subgroup analysis also showed that high MLR was an effective predictor of 90-day mortality in T2DM patients with CKD under various specific conditions. MLR is superior to other conventional markers because it is cheap, effective, and easy to obtain. Meanwhile, MLR is more stable and conducive to clinical application compared with a single indicator.

In our study, several limitations were observed as follows: First, A single-centric retrospective study had the possibility of selection bias. Second, some unrecorded clinical information, such as the condition before entered to the ICU, may affect the outcome. Third, there were uncontrollable confounding factors affecting monocyte and lymphocyte counts, such as the use of drug and unspecified comorbidities. Finally, the underlying mechanism between MLR and prognosis could not be determined. Therefore, a large multicenter prospective study should be designed to confirm the above results and further study the mechanism.

## Conclusion

In this study, we demonstrated that higher MLR was significantly associated with an increased risk of 90-day all-cause mortality in T2DM patients with CKD in ICU. MLR could be served as a predictive and effective marker due to its inexpensive and reliability.

## Data Availability

Original data used in this study is from the MIMIC-III database: MIMIC III (https://physionet.org/content/mimiciii/1.4/, version 1.4). The author (S.L.) obtained access to this database (certification number: 42883491) and was responsible for extracting the data. If needed, related data can be provided by contacting G.H. and S.L.
